# KLF4 defines the efficacy of the epidermal growth factor receptor inhibitor, erlotinib, in triple-negative breast cancer cells by repressing the *EGFR* gene

**DOI:** 10.1186/s13058-020-01305-7

**Published:** 2020-06-18

**Authors:** Melyssa S. Roberts, Lindsey J. Anstine, Viviane S. Finke, Benjamin L. Bryson, Bryan M. Webb, Kristen L. Weber-Bonk, Darcie D. Seachrist, Parth R. Majmudar, Ruth A. Keri

**Affiliations:** 1 Department of Pharmacology, School of Medicine, Case Western Reserve University, Cleveland, OH 44106 USA; 2 Department of Biochemistry, School of Medicine, Case Western Reserve University, Cleveland, OH 44106 USA; 3 Department of Genetics and Genome Sciences and Division of General Medical Sciences-Oncology, Case Western Reserve University, Cleveland, OH 44106 USA

**Keywords:** Triple-negative breast cancer (TNBC), Epidermal growth factor receptor (EGFR), Krüppel-like factor 4 (KLF4), Erlotinib

## Abstract

**Background:**

Triple-negative breast cancer (TNBC) is characterized by high rates of recurrence and poor overall survival. This is due, in part, to a deficiency of targeted therapies, making it essential to identify therapeutically targetable driver pathways of this disease. While epidermal growth factor receptor (EGFR) is expressed in 60% of TNBCs and drives disease progression, attempts to inhibit EGFR in unselected TNBC patients have had a marginal impact on outcomes. Hence, we sought to identify the mechanisms that dictate EGFR expression and inhibitor response to provide a path for improving the utility of these drugs. In this regard, the majority of TNBCs express low levels of the transcription factor, Krüppel-like factor 4 (KLF4), while a small subset is associated with high expression. KLF4 and EGFR have also been reported to have opposing actions in TNBC. Thus, we tested whether KLF4 controls the expression of EGFR and cellular response to its pharmacological inhibition.

**Methods:**

KLF4 was transiently overexpressed in MDA-MB-231 and MDA-MB-468 cells or silenced in MCF10A cells. Migration and invasion were assessed using modified Boyden chamber assays, and proliferation was measured by EdU incorporation. Candidate downstream targets of KLF4, including EGFR, were identified using reverse phase protein arrays of MDA-MB-231 cells following enforced KLF4 expression. The ability of KLF4 to suppress EGFR gene and protein expression and downstream signaling was assessed by RT-PCR and western blot, respectively. ChIP-PCR confirmed KLF4 binding to the EGFR promoter. Response to erlotinib in the context of KLF4 overexpression or silencing was assessed using cell number and dose-response curves.

**Results:**

We report that KLF4 is a major determinant of EGFR expression and activity in TNBC cells. KLF4 represses transcription of the *EGFR* gene, leading to reduced levels of total EGFR, its activated/phosphorylated form (pEGFR), and its downstream signaling intermediates. Moreover, KLF4 suppression of EGFR is a necessary intermediary step for KLF4 to inhibit aggressive TNBC phenotypes. Most importantly, KLF4 dictates the sensitivity of TNBC cells to erlotinib, an FDA-approved inhibitor of EGFR.

**Conclusions:**

KLF4 is a major regulator of the efficacy of EGFR inhibitors in TNBC cells that may underlie the variable effectiveness of such drugs in patients.

## Introduction

Triple-negative breast cancer (TNBC) comprises ~ 20% of all breast malignancies. This histologic subtype lacks the expression of progesterone and estrogen receptors and does not display amplification of the human epidermal growth factor receptor 2 (HER2) gene. Given the lack of these targetable receptors, chemotherapy is the standard treatment for TNBC. In 2019, atezolizumab, a PD-L1 inhibitor, was approved for the treatment of advanced or metastatic TNBC in combination with nab-paclitaxel [1]. As this therapy achieved an objective response in 53% of treated patients, approximately half of the patient population remains without a targeted therapeutic option. Likewise, PARP inhibitors have also recently received FDA approval, but these are limited to patients with germline BRCA1 mutations. In addition to lacking targeted therapies, TNBC is the most aggressive breast cancer subtype, leading to a poor patient prognosis, as it is highly metastatic and invasive [2, 3]. Once metastasis occurs, the median overall survival rate is only 12 months [4, 5]. This is compared to non-metastatic TNBC patients, whose median overall survival rate is 3.55 years [6]. Discerning the molecular mechanisms that underlie TNBC aggressive phenotypes will be essential for improving therapeutic approaches and, hence, patient outcomes.

Up to 76% of metastatic breast carcinomas overexpress the epidermal growth factor receptor (EGFR), and this is associated with shorter patient survival [7–9]. EGFR is a receptor tyrosine kinase that is a key driver of TNBC that functions by stimulating diverse cancer-promoting signaling pathways such as Ras-Raf-MEK-ERK, PI3K-AKT-mTOR, and Src-STAT3 [10]. Activation of such pathways subsequently promotes proliferation, migration, motility, invasion, and survival of breast cancer cells [11–15]. These characteristics make EGFR an attractive therapeutic target to combat TNBC.

Currently, there are two classes of drugs that target EGFR: monoclonal antibodies (mAbs) and tyrosine kinase inhibitors (TKIs). mAbs bind to the extracellular domain of EGFR resulting in competitive antagonism and preventing the activation of downstream signaling pathways. However, mAbs have displayed limited efficacy in breast cancer clinical trials [16]. TKIs that target EGFR, including erlotinib, gefitinib, afatinib, and lapatinib, function by competitively binding the ATP site within the catalytic domain and preventing the phosphorylation/activation of EGFR targets including AKT and ERK [17]. EGFR inhibitors have been particularly successful in non-small cell lung cancer, an EGFR-driven disease, and they are now the standard of care for patients with an EGFR-sensitizing mutation [18]. Launching from this success, several clinical trials have been completed in breast cancer patients using EGFR-targeted TKIs, either as monotherapy or in addition to chemotherapy [19–24]. While early clinical trials seemed promising, further studies have proven disappointing due to low efficacy when evaluating unselected breast cancer patient cohorts. An erlotinib trial for patients with locally advanced or metastatic breast cancer had low overall success. However, it is important to note that 8% of patients did respond to this drug [21]. The efficacy of combining erlotinib with the cytotoxic drug, bendamustine, was also assessed in TNBC patients [22]. Again, only 9% of patients experienced a partial response. These data suggest that, while not effective in most patients, there are breast cancers that can respond to EGFR inhibition. Discovering the basis for response could lead to the effective use of EGFR-targeting therapies in a subset of breast cancer patients. We postulated that factors controlling the expression or activity of EGFR may dictate the response.

Krüppel-like factor 4 (KLF4) is a zinc finger transcription factor that exhibits dichotomous activity in a variety of processes during development and cancer. In breast cancer, KLF4 can have both tumor suppressive and oncogenic functions [25, 26]. Hence, the actions of KLF4 are highly dependent upon cell and tissue context, with its specific roles in breast cancer remaining controversial. Aligning with its role as a pluripotency transcription factor, KLF4 can stimulate stem cell properties in breast cancer cells [27, 28]. However, KLF4 has also been reported to be a metastasis suppressor in breast cancer where it represses proliferation, migration, and invasion and promotes cell cycle arrest and apoptosis [25, 26, 28–31]. KLF4 is also a major regulator of EMT, a process known to promote metastasis. This involves transcriptional repression of mesenchymal genes and activation of epithelial genes, including E-cadherin, resulting in the inhibition of in vitro metastatic phenotypes in breast cancer cells [29, 32]. Moreover, in vivo analyses have shown that KLF4 suppresses tumorigenicity and inhibits primary tumor growth and metastases in xenograft models [26, 33].

It has previously been reported that 68% of primary TNBCs have low KLF4 expression, while 32% highly express KLF4 [31]. It is unclear whether the tumors that express high levels of KLF4 may display distinct phenotypes from those with low expression, including potential differential response to therapies. Herein, we identify KLF4 as a critical regulator of EGFR and of erlotinib response in TNBC cells. In light of the controversial functions reported for KLF4 in breast cancer, we confirmed that it inhibits several phenotypes associated with aggressive disease including proliferation, invasion, and migration. We further found that the ability of KLF4 to prevent these properties is due to its repression of *EGFR* gene expression. Most importantly, we found that the inhibition of EGFR by KLF4 modulates TNBC cell responsiveness to EGFR inhibitors such as erlotinib.

## Methods

### Cell culture and reagents

All cell lines were acquired from the American Type Culture Collection (ATCC) and were cultured at 37 °C with 5% CO_2_. MDA-MB-231 and MDA-MB-468 cell lines were maintained in RPMI-1640 supplemented with 10% FBS. MCF10A cells were cultured in DMEM F-12 supplemented with cholera toxin, 1% l-glutamine, hydrocortisone, insulin, 5% horse serum, and epidermal growth factor. All cell lines were tested monthly for *Mycoplasma pulmonis* and *Mycoplasma* spp. (Bimake, B39032).

MDA-MB-468 and MDA-MB-231 cells were infected with empty vector adenovirus control (AdGFP) or adenovirus overexpressing KLF4 (AdKLF4) for 24 h as previously described [29]. Transient mRNA silencing was completed using 100 nM non-targeting siRNA (Dharmacon, D-001810-02) or siRNA targeting *KLF4* (L-005089-00) or *EGFR* (L-003114-00) with Lipofectamine 2000 (Invitrogen, 11668-492 027) in Opti-MEM media (Invitrogen, 31985088) for 6 h.

Erlotinib (Selleckchem, S1023) was dissolved in dimethyl sulfoxide (DMSO). For dose-response curves, cells were treated with the indicated concentration of drug for 3 days. Cells were then trypsinized, and viable cells were counted by trypan blue exclusion on a Countess II FL (Thermo Fisher, AMQAF1000). For all assays with drug treatment and siRNA interference or adenoviral infection, cells were transfected/infected with siRNA/adenovirus for 6/24 h after which they were maintained in complete media for 24 h. Erlotinib was then added, and cells were counted 3 days later.

### RNA analysis

RNA analysis was performed as previously reported [34] using TRIzol Reagent (Ambion, 15596018) for RNA isolation. SuperScript II reverse transcriptase (Invitrogen, 18064-014) was used to perform reverse transcription. *KLF4* (Hs00358836_m1), *EGFR* (Hs01076090_m1), and *GAPDH* (Hs02758991_g1) TaqMan Gene Expression assays (Thermo Fisher) were used to perform quantitative real-time PCR on an Applied Biosystems StepOnePlus Real-Time PCR System.

### Western blots

Cells were lysed using RIPA buffer as previously described [35]. Protein concentrations were quantified using the Bradford assay (Bio-Rad, 5000006). Protein lysate (50 μg) was resolved using SDS-PAGE before transferring to a PVDF membrane (Millipore, 1PFL00010). Blots were blocked for 1 h in 5% milk/TBS, then rinsed in water, incubated for 5 min in 5 mL of REVERT Total Protein Stain (LI-COR, 926-11010), and washed (6.7% glacial acetic acid, 30% methanol, and water) twice for 30 s. Total protein staining was utilized as opposed to β-actin due to research showing greater accuracy of total protein staining as a normalization control [36–38]. Blots were imaged for total protein using the Odyssey FC Imaging System (LI-COR). The REVERT stain was removed with reversal solution (1 M NaOH, methanol, and water) for 5 min, and the blots were incubated overnight at 4 °C in primary antibody diluted in 5% BSA/TBST at concentrations of 1:1000. Primary antibodies were KLF4 (rabbit, Cell Signaling, 12173), tEGFR (rabbit, Cell Signaling, 4267), pEGFR Y1068 (rabbit, Cell Signaling, 3777), pEGFR (rabbit, Cell Signaling, 4407), tAKT (mouse, Cell Signaling, 2920), pAKT (rabbit, Cell Signaling, 4060), tERK1/2 (rabbit, Cell Signaling, 4695), and pERK1/2 Y202/Y204 (mouse, Cell Signaling, 9106). Blots were incubated in secondary antibody (LI-COR, 925-32211) at a 1:20,000 dilution in 5% milk/TBST for 1 h. They were then imaged on the Odyssey FC Imaging System. The proteins were quantified using Image Studio Lite Ver. 5.2 and normalized versus their respective total protein.

### Gene-specific chromatin immunoprecipitation

ChIP-PCR was performed in MCF10A cells as previously reported [29]. A KLF4-specific antibody (Santa Cruz, sc-166238) or control mouse IgG (Sigma, I5281) was used to immunoprecipitate the chromatin. Promoter-specific primer sequences are listed in Table S1.

### 5-Ethynyl-2′-deoxyuridine incorporation

MCF10A cells were transfected with siNS, siKLF4, siEGFR, or siKLF4+siEGFR. After 2 days, 10 μM 5-ethynyl-2′-deoxyuridine (EdU; Thermo Fisher, C10339) was added according to the manufacturer’s protocol. After a 16-h incubation, VECTASHIELD HardSet Mounting Medium with DAPI (Vector Labs, H-1500) was used to counterstain the nuclei. EdU-positive cells were counted using an inverted Leica fluorescence microscope and were expressed relative to the total cell number.

### Reverse phase protein array

MDA-MB-231 cells were infected with AdGFP or AdKLF4 for 24 h. After 3 days, cells were lysed using lysis buffer (1% Triton X-100; 50 mM HEPES, pH 7.4; 150 mM NaCl; 1.5 mM MgCl2; 1 mM EGTA; 100 mM NaF; 10 mM Na pyrophosphate; 1 mM Na3VO4; 10% glycerol; and Protease Inhibitor Cocktail (Sigma, P8340-5ML) and incubated on ice for 20 min. Cells were scraped and centrifuged at 14,000 rpm for 10 min at 4 °C. Protein concentration was calculated using a Bradford assay and adjusted to 1.5 μg/μl. The reverse phase protein array (RPPA) was then probed and evaluated as previously described [39, 40]. Briefly, cell lysates were mixed 3:1 with sample buffer (40% glycerol; 8% SDS; 0.25 M Tris-HCL, pH 6.8; and 10% beta-mercaptoethanol) and boiled for 5 min. Samples were then serially diluted twofold for five dilutions and arrayed on nitrocellulose-coated slides to produce sample spots. These were then probed with antibodies by a tyramide-based signal amplification approach and visualized by DAB colorimetric reaction to produce stained slides. The slides were scanned on a Huron TissueScope scanner, and sample spot densities were quantified by Array-Pro Analyzer 6.3. The data was fitted to a standard curve, and relative protein levels were designated as log2 values and normalized for protein loading. The interactome analysis was completed using STRING version 11.0 on proteins downregulated after AdKLF4 with a fold change greater than 2-fold.

### Transwell migration and invasion assays

Assays were performed as previously described [29]. Briefly, 100,000 cells were seeded in serum-free media onto transwell supports (Corning, 3422) or invasion chambers (Corning, 354480). Cells were allowed to migrate or invade toward complete medium for 6 or 16 h, respectively. Five (× 20) fields per transwell support were counted.

### Hoechst staining

Three days after cells were transfected with siNS, siKLF4, siEGFR, or siKLF4+siEGFR, they were stained with 10 μM Hoechst (Thermo Scientific, 62249) for 10 min at RT. Cells with and without pyknotic nuclei were quantified to calculate the percent of apoptotic cells.

### Statistical methods

Statistical analyses were performed using two-tailed Student’s *t* test or nonlinear regression analysis when appropriate. Significance was concluded if the *p* value was less than 0.05. Unless otherwise noted, all data are represented as the mean with the error bars denoting the standard deviation of three independent experiments completed in triplicate.

## Results

### KLF4 represses migration, invasion, and cell growth of breast epithelial cells

**Fig. 1:**
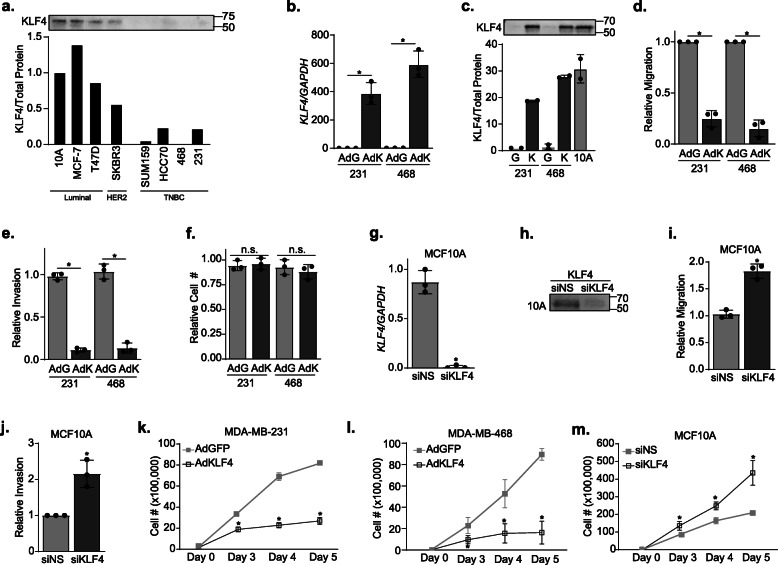
KLF4 represses migration, invasion, and long-term cell growth of breast epithelial cells. **a** Western blot analysis and quantitation of KLF4 expression across eight different breast cell lines: MCF10A (10A), MCF-7, T47D, SKBR3, SUM159, HCC70, MDA-MB-468 (468), and MDA-MB-231 (231). **b** RT-qPCR analysis of KLF4 expression levels in MDA-MB-231 and MDA-MB-468 cell lines 3 days after infection with AdGFP (AdG) or AdKLF4 (AdK). **c** Western blot analysis and quantitation showing KLF4 expression in MDA-MB-231 and MDA-MB-468 following infection with AdGFP (G) or AdKLF4 (K) and compared to endogenous levels in MCF-10A cells. Two biological replicates were performed. Cells were infected with AdG or AdK for 2 days and then allowed to **d** migrate for 6 h or **e** invade into Matrigel for 24 h before they were stained and counted. **f** MDA-MB-231 and MDA-468 cells were infected with AdG or AdK and plated. After 24 h, they were counted using trypan blue exclusion. **g** RT-qPCR and **h** western blot analysis of the KLF4 expression in MCF10A cells 3 days post-infection with a non-targeting siRNA (siNS) or siRNA targeting KLF4 (siKLF4). Cells were infected with siNS or siKLF4 and allowed to **i** migrate for 6 h or **j** invade for 24 h before they were stained and counted. Cells were **k**, **l** infected with AdG or AdK or **m** transfected with siNS or siKLF4, and the cell number was counted at days 3–5 using trypan blue exclusion assay. Relative values were normalized versus the control, and error bars represent the standard deviation. Experiments were performed three independent times and in triplicate with *p < 0.05

Given the disparate reports on the oncogenic/tumor-suppressive functions of KLF4 in breast epithelium, we first sought to confirm the previous findings on the impact of KLF4 on proliferation, migration, and invasion across multiple breast epithelial cell lines [26, 29, 31]. We examined the pattern of expression of KLF4 in seven different breast cancer cell lines as well as a non-transformed mammary epithelial cell line (MCF10A). KLF4 was highly expressed in the luminal and non-transformed lines. In addition, this analysis revealed that KLF4 expression was low in TNBC cell lines compared to non-TNBC cell lines (Fig. 1a, total protein image in Fig. S1A). To determine if KLF4 inhibits pro-metastatic properties associated with TNBC, we assessed the impact of modulating KLF4 levels on migration, invasion, and cell number in multiple cell lines. We overexpressed KLF4 in two TNBC cell lines, MDA-MB-231 and MDA-MB-468, both of which have low endogenous KLF4 expression, using an adenoviral overexpression vector (AdKLF4) or an adenoviral vector expressing GFP (AdGFP) as a negative control (Fig. 1b, qRT-PCR; Fig. 1c, western blot; total protein image in Fig. S1B). Notably, the degree of KLF4 overexpression as a result of AdKLF4 infection in these cells was similar to the levels of KLF4 observed in MCF10A cells, indicating that the expression levels were within the endogenous range of non-transformed cells. To assess the impact of enforced KLF4 expression on TNBC cell migration, infected cells were seeded in modified Boyden chambers and allowed to migrate for 6 h using serum as the chemoattractant. Overexpression of KLF4 (Fig. 1b, c) decreased the number of MDA-MB-231 and MDA-MB-468 cells that migrated by 75% and 85%, respectively (Fig. 1d). The impact of KLF4 expression on invasion was also evaluated. The same cell lines were infected with KLF4 or GFP-expressing adenovirus as described above and then seeded into Matrigel-coated invasion chambers. Cells that invaded the matrigel were quantified 24 h later. Enforced expression of KLF4 had an even greater impact on invasion, decreasing the number of MDA-MB-231 and MDA-MB-468 cells that could invade through the matrix by 89% and 87%, respectively (Fig. 1e). To ensure these changes were not simply due to the decreased proliferation during the time course of this experiment, we measured the cell number 24 h after plating. No changes in cell number were observed at this short time point; thus, the results observed in the invasion studies were not confounded by a decrease in cell number (Fig. 1f).

Complementary experiments were then conducted to assess the impact of silencing KLF4 on migration and invasion. We were unable to determine the impact of KLF4 silencing in the MDA-MB-231 and MDA-MB-468 cell lines because their endogenous expression of KLF4 is very low. Instead, we utilized the non-transformed MCF10A cell line which expresses higher levels of KLF4 (Fig. 1a) but has a similar transcriptome as TNBC cell lines [41]. We transfected non-targeting siRNA (siNS) or siRNA targeting KLF4 (siKLF4) (Fig. 1g, h, total protein image in Fig. S1C) and found that suppression of KLF4 nearly doubled the number of cells that migrated toward the serum (Fig. 1i). Moreover, silencing KLF4 also significantly increased the number of invading cells (Fig. 1j).

We then asked if modulating KLF4 during a more extended time frame would impact cell growth. We overexpressed KLF4 in MDA-MB-231 and MDA-MB-468 cells by infection with AdGFP or AdKLF4 and quantified the number of live cells 3, 4, and 5 days thereafter. Beginning 3 days after plating, KLF4 overexpression repressed TNBC cell growth (Fig. 1k, l). In the complementary experiment, silencing KLF4 in MCF10A cells caused a significant increase in cell number beginning at 3 days post-transfection with siKLF4 compared to the non-silencing control (siNS) (Fig. 1m). Together, these data affirm that KLF4 represses a broad range of metastasis-associated phenotypes including cell growth, migration, and invasion in tumorigenic and non-tumorigenic breast epithelial cell lines.

### Identification of a KLF4-regulated protein signature reveals EGFR as a downstream target

**Fig. 2:**
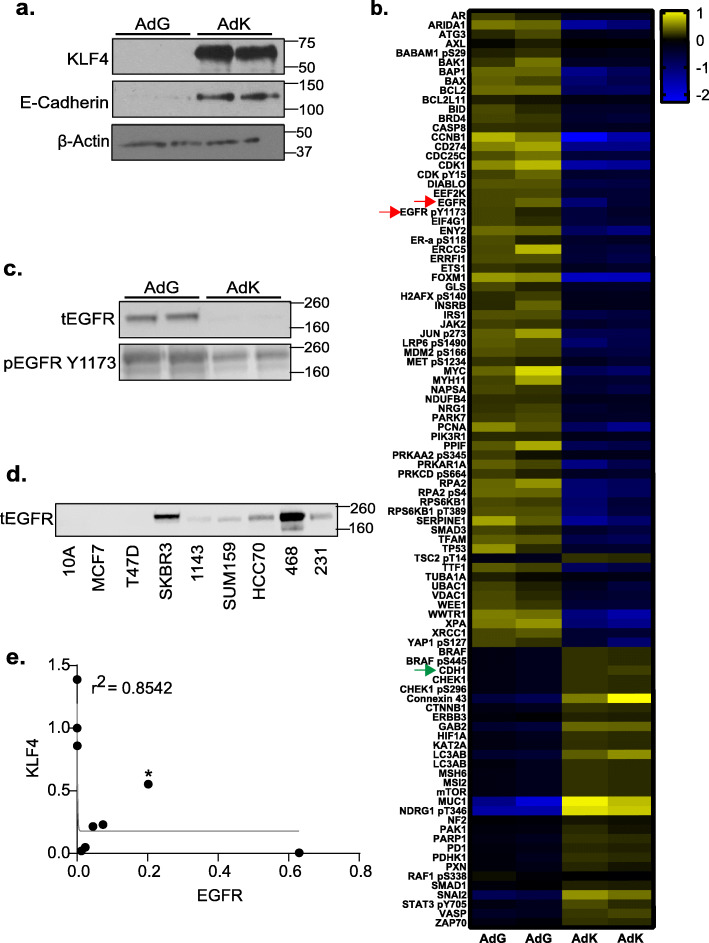
Identification of a KLF4-regulated protein signature reveals EGFR as a downstream target. **a** Western blot confirming the upregulation of KLF4 and its downstream target, E-cadherin, in MDA-MB-231 cells after AdGFP or AdKLF4 infection. **b** Reverse phase protein array heatmap showing differentially expressed genes after AdGFP or AdKLF4 infection in MDA-MB-231 cells. This analysis was completed as one experiment in duplicate. Proteins whose expression fold change was significantly different (*p* < 0.05) between AdKLF4 and AdGFP are shown. Red arrows indicate suppression of EGFR and phosphorylated EGFR by KLF4 overexpression while the green arrow identifies upregulated E-cadherin following KLF4 overexpression. **c** Western blot confirming EGFR repression in MDA-MB-231 cells infected with AdKLF4 compared to those infected with AdGFP. **d** Western blot analysis of EGFR expression across eight breast epithelial cell lines: MCF10A (10A), MCF7, T47D, SKBR3, HCC1143 (1143), SUM159, HCC70, MDA-MB-468 (468), and MDA-MB-231 (231). **e** Inverse correlation between KLF4 and EGFR protein in the eight breast epithelial cell lines, *r*^2^ = 0.8542. *SKBR3 cell line

To further elucidate the mechanism by which KLF4 represses aggressive phenotypes in TNBC, we sought to identify downstream protein effectors that mediate its actions on growth, migration, and invasion. While previous studies have reported the use of microarrays to identify KLF4 gene targets in corneal, rectal carcinoma, and non-transformed mammary epithelial cell lines [32, 42, 43], no reports to date have examined the impact of KLF4 on the expressed proteome in breast cancer cells. To discover downstream protein targets of KLF4 in breast cancer, we examined the changes in protein expression and phosphorylation using a reverse phase protein array (RPPA) of extracts from MDA-MB-231 cells overexpressing KLF4 as a result of AdKLF4 infection compared to cells infected with AdGFP. Two days after infection, protein was harvested and expression of KLF4 and its previously reported target, E-cadherin (*CDH1*), were confirmed (Fig. 2a) [29]. Using the RPPA core at MD Anderson Cancer Center, we analyzed the expression of 297 proteins and/or their phosphorylated forms. This yielded 113 downregulated proteins and 17 upregulated proteins whose expression changed by ≥ 1.5 fold (Fig. 2b). Those with the greatest changes are listed in Supplemental Figure 2A-B. Confirming the validity of the RPPA, CDH1 protein expression was elevated in response to KLF4 overexpression. This analysis further revealed that both total EGFR and its phosphorylated/activated isoform (pEGFR, Y1173) were reduced with KLF4 overexpression. The suppression of total and phosphorylated EGFR levels by KLF4 was further validated using western blots of distinct lysates from KLF4 overexpressing cells (Fig. 2c, total protein image in Fig. S2C).

Notably, activated EGFR inversely regulates the same metastatic steps as KLF4. For example, EGFR promotes MDA-MB-231 migration, while KLF4 represses it [13, 29]. Additionally, EGFR and pEGFR proteins are highly expressed in invasive breast cancer and are associated with worse overall survival rates [14], while high KLF4 is associated with improved survival outcomes. Moreover, low KLF4 expression is correlated with increased recurrence, and its expression is decreased in breast cancers compared to adjacent normal parenchyma [31, 33]. Additionally, we found EGFR to be at the center of interactions with other proteins found to be changed in the RPPA (Fig. S2D). These seemingly opposing functions prompted us to determine if EGFR protein expression is inversely correlated with KLF4 in the eight breast epithelial cell lines we interrogated for KLF4 expression in Fig. 1. As expected, EGFR protein expression is highest in the TNBC cell lines compared to those from other breast cancer subtypes (Fig. 2d, S1A). Moreover, KLF4 and EGFR protein expression are generally mutually exclusive in the breast cancer cell lines we examined, suggesting that a threshold level of endogenous KLF4 may suppress the expression of EGFR in breast cancer cells (Fig. 2e). One exception to this is the luminal SKBR3 cell line, which may have alternate signaling mechanisms due to its expression of HER2.

### KLF4 represses the EGFR signaling pathway

**Fig. 3:**
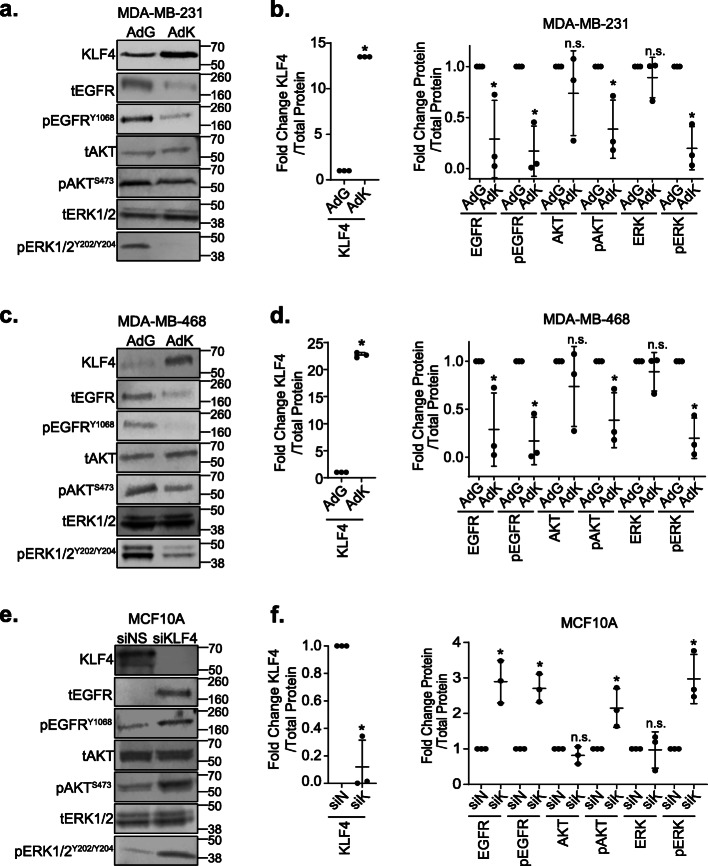
KLF4 represses the EGFR signaling pathway. **a** Western blot analysis of KLF4, tEGFR, pEGFR (Y1068), tAKT, pAKT (S473), tERK1/2, and pERK1/2 (Y202/Y204) protein levels 3 days after AdGFP or AdKLF4 infection of MDA-MB-231 cells. **b** Graph depicting western blot quantification. All protein levels were normalized to total protein in the lane to control for loading, and phosphorylated proteins were normalized to their respective unphosphorylated proteins. AdKLF4 lanes were then expressed relative to AdGFP and are graphed with the horizontal bars indicating the median of the values of three replicate blots. Each dot represents the mean value from a different experiment, *p < 0.05. **c**, **d** Similar to **a** and **b**, but with lysates from infected MDA-MB-468 cells. **e** Western blot analysis of the same proteins in **a**, collected 3 days after transfection of MCF10A cells with siNS or siKLF4. **f** Quantitation and analysis of protein levels were completed as described in **b**. All western blots were completed in three independent experiments, each in triplicate, *p < 0.05

EGF binding to EGFR leads to receptor dimerization and trans-phosphorylation of several tyrosine residues. This promotes the recruitment of multiple effector proteins that induce downstream signaling cascades such as MEK-ERK and PI3K-AKT and activation of various metastasis-associated cellular phenotypes. To determine if the impact of KLF4 on EGFR expression was translated to a change in EGFR downstream signaling, we examined the activation status of AKT and ERK following KLF4 manipulation. As expected, overexpression of KLF4 in MDA-MB-231 cells caused a decrease in both total and phosphorylated EGFR (Y1068) (Fig. 3a, b, total protein image in Fig. S3A). Likewise, phosphorylation of AKT (S473) and ERK1/2 (Y202/Y204) was significantly reduced when KLF4 was overexpressed while there was no change in total AKT and total ERK1/2 protein expression. KLF4 signaling through EGFR may be occurring largely through ERK as opposed to AKT, as phosphorylated ERK (pERK) was decreased to a greater extent than phosphorylated AKT (pAKT). Similar effects of KLF4 overexpression were observed in MDA-MB-468 cells (Fig. 3c, d, total protein image in Fig. S3B), indicating that signaling changes that occur in response to enforced KLF4 expression are not unique to the MDA-MB-231 cell line. In a complementary study, we silenced endogenous KLF4 in MCF10A cells with siNS or siKLF4 and, as anticipated, found that suppressing KLF4 expression results in an increase in total EGFR and pEGFR. Likewise, pAKT and pERK were increased while total AKT and ERK protein levels did not change (Fig. 3e, f, S3C). Together, these data indicate that KLF4 regulates the amount of total EGFR that is expressed. As a consequence, this subsequently alters downstream EGFR signaling to both the AKT and ERK pathways.

### KLF4 represses transcription of the EGFR gene

**Fig. 4:**
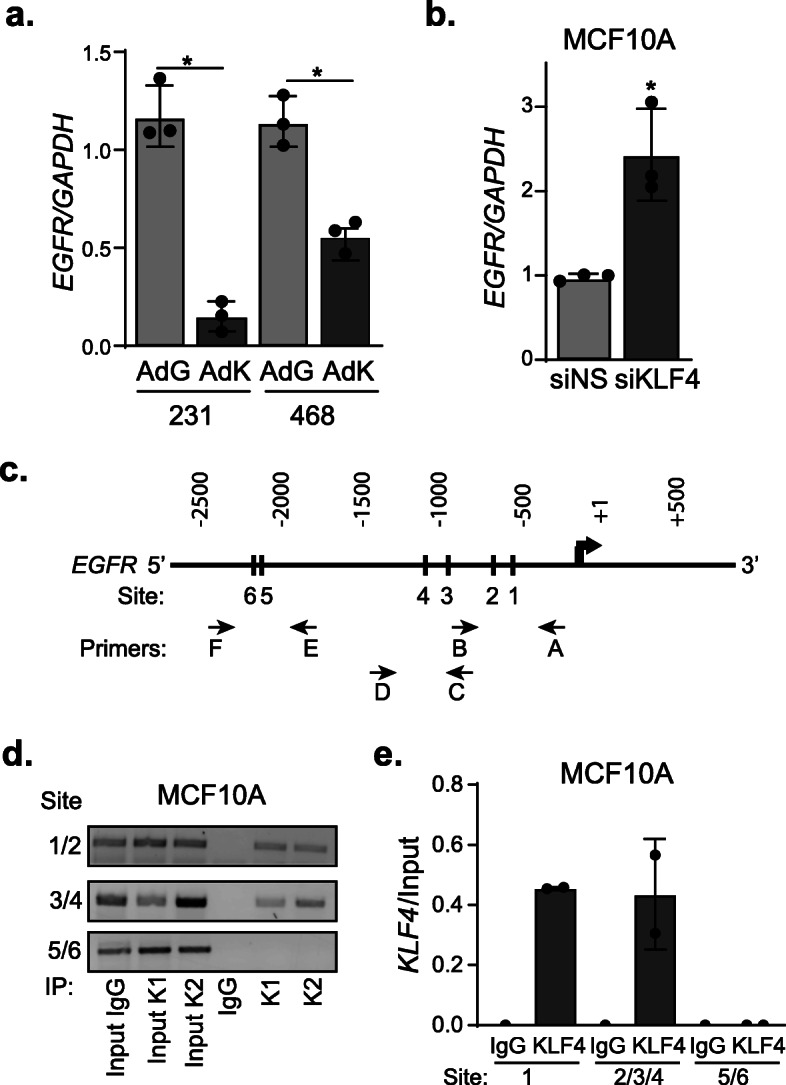
KLF4 represses transcription of the EGFR gene. **a** RT-qPCR quantitation of EGFR mRNA levels following infection of MDA-MB-231 (231) and MDA-MB-468 (468) cells with AdGFP or AdKLF4. **b** RT-qPCR analysis of EGFR mRNA after silencing KLF4 in MCF10A cells. **c** Schematic of *EGFR* promoter-specific sites (1–6) used to detect KLF4 binding by ChIP-PCR. Three primer sets (A/B, C/D, and E/F) were used as indicated in the schematic and labels. Primers are listed in Table S1. **d** Gene-specific ChIP-PCR gel of MCF10A cells assessing the binding of KLF4 protein to the EGFR gene locus (hg19) where K1 and K2 are technical replicates for KLF4 immunoprecipitation. **e** Quantitation of **d** relative to the input. **a**, **b** Completed in three independent experiments in triplicate with *p < 0.05. **d**, **e** Completed in two independent experiments in duplicate

As a transcriptional modulator, the most direct way for KLF4 to regulate EGFR protein levels is by repressing *EGFR* transcription. Indeed, enforced expression of KLF4 in both MDA-MB-231 and MDA-MB-468 cell lines resulted in decreased *EGFR* mRNA levels (Fig. 4a). Conversely, KLF4 silencing in MCF10A cells increased *EGFR* mRNA expression (Fig. 4b). To determine if KLF4 directly binds to the *EGFR* gene, we conducted gene-specific chromatin immunoprecipitation (ChIP) for endogenous KLF4 in MCF10A cells. This was followed by PCR for six KLF4 consensus sites within the *EGFR* promoter regulatory region (Supplemental Table 1). This revealed that KLF4 binds to multiple locations in the *EGFR* promoter (Fig. 4c–e), indicating that KLF4 represses *EGFR* gene expression.

### Repression of EGFR is an obligatory intermediate step for KLF4 to inhibit aggressive breast cancer phenotypes

**Fig. 5:**
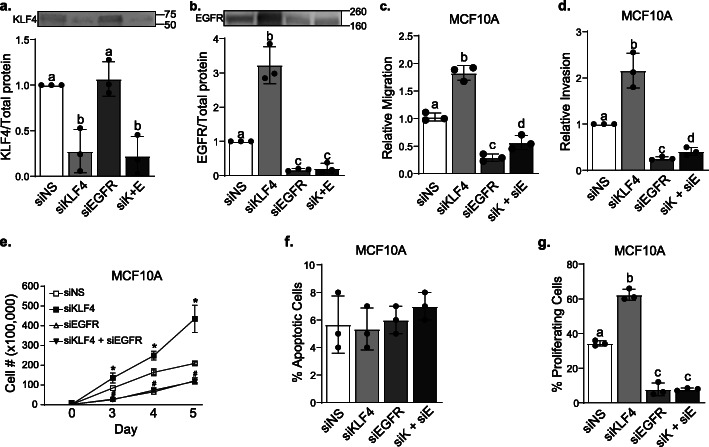
Repression of EGFR is an obligatory intermediate step for KLF4 to inhibit aggressive breast cancer phenotypes. MCF10A cells were transfected with siNS, siKLF4, siEGFR, or siKLF4+siEGFR, and western blots were performed to confirm **a** KLF4 and **b** EGFR silencing. Cells were then allowed to **c** migrate for 6 h or **d** invade for 16 h before they were stained and counted. **e** MCF10A cells were transfected with siNS, siKLF4, siEGFR, or siKLF4+siEGFR, and cell number was counted at days 3–5 using trypan blue exclusion assay, *p < 0.05. **f** Apoptotic MCF10A cells were assessed using Hoechst stain 3 days after transfection. **g** EdU staining was used to quantify the number of proliferating MCF10A cells 3 days post-transfection. For all data, relative values were normalized versus the control, and error bars represent the standard deviation. Bars with distinct letters above them are significantly different from one another (*p* < 0.05). Experiments were performed three independent times in triplicate

As indicated above, the TNBC phenotypes associated with metastasis are induced by EGFR and repressed by KLF4. To determine if the suppression of EGFR is necessary for KLF4 to restrain proliferation, migration, and invasion, we prevented the induction of EGFR expression that occurs when KLF4 is silenced in MCF10A cells by co-transfecting with an EGFR-targeted siRNA (Fig 5a, b, total protein image in S4A-B). Consistent with our previous findings, silencing KLF4 alone (Fig. 5a) led to an increase in migration (Fig. 5c) and invasion (Fig. 5d) compared to cells transfected with the non-silencing control siRNA (siNS). In contrast, silencing EGFR alone (Fig. 5b) decreased migration and invasion (Fig. 5c, d). Most importantly, blocking the induction of EGFR that occurs when KLF4 is silenced largely prevented the increase in migration and invasion that is observed when just KLF4 is lost (Fig. 5c, d). Hence, the induction of EGFR expression is an important factor for the increase in migration and invasion that is observed with the loss of KLF4. We did note that KLF4 silencing could still induce a small increase in migration and invasion even when EGFR expression was reduced. This could be due to the lack of complete elimination of EGFR expression or to additional signaling pathways controlled by KLF4 that also facilitate these properties.

To determine if the impact of KLF4 on cell growth is also dependent upon its repression of EGFR expression, we silenced each individually or in combination (Fig. 5a, b, S4A-B) and measured the number of live cells for 5 days. Strikingly, cell growth after dual suppression of KLF4 and EGFR was identical to that of EGFR silencing alone (Fig. 5e). This suggests that KLF4 regulation of cell growth is entirely dependent on its regulation of EGFR. To determine if the changes observed in cell number are due to alterations in apoptosis or proliferation, we completed both Hoechst staining and EdU incorporation assays, respectively. No difference in apoptosis was observed after the individual or combined suppression of KLF4 and/or EGFR (Fig. 5f), indicating that cell death does not underlie the changes in cell growth. In contrast, silencing KLF4 expression increased the percentage of EdU-positive cells while EGFR silencing decreased this percentage (Fig. 5g), indicating that they inversely impact proliferation. After simultaneous repression of EGFR and KLF4, the percent of EdU-positive cells decreased to the same extent as observed with EGFR silencing alone. Taken together, these results indicate that the ability of KLF4 to suppress proliferation, migration, or invasion is either partially or fully dependent upon its repression of EGFR expression and is phenotype-specific.

### KLF4 expression dictates sensitivity to pharmacological inhibition of EGFR

**Fig. 6:**
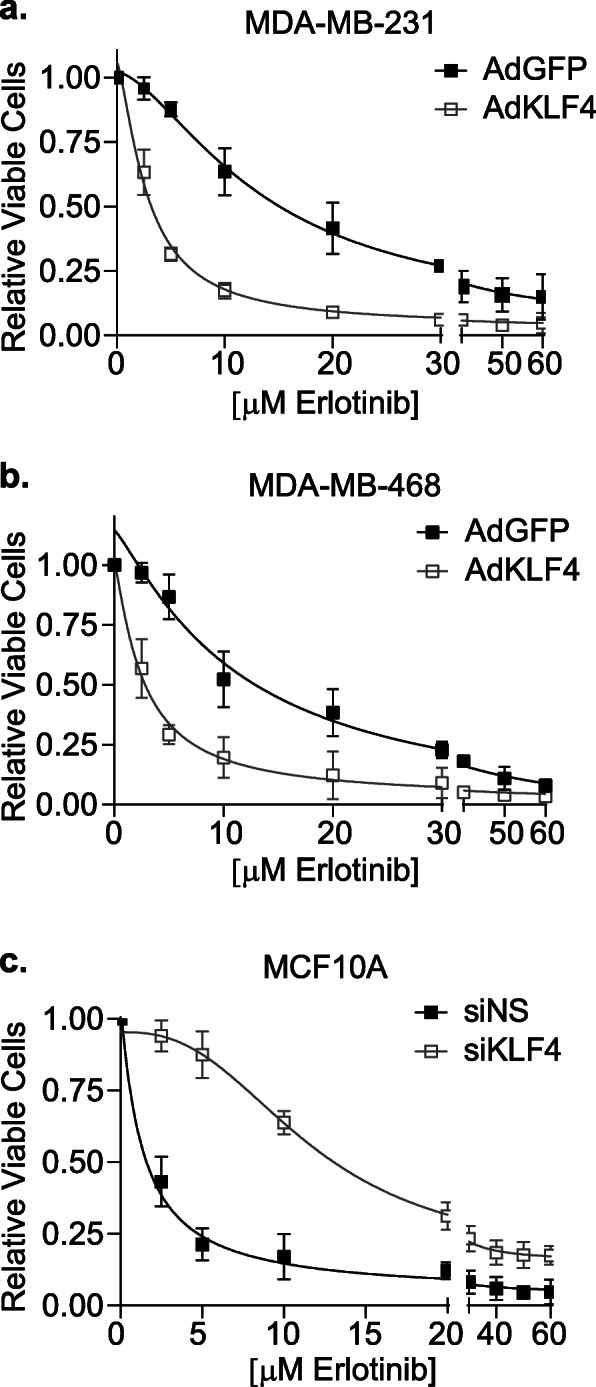
KLF4 expression dictates sensitivity to pharmacological inhibition of EGFR. **a** MDA-MB-231 and **b** MDA-MB-468 cells were infected with AdGFP or AdKLF4, and 2 days later, treated with 0–60 μM erlotinib for 3 days. The live cells were counted using trypan blue exclusion assay. Responses were normalized to effects observed with no drug for both AdGFP- and AdKLF4-infected cells. **c** MCF10A cells were transfected with siNS or siKLF4, and after 1 day, were treated with 0–60 μM erlotinib for 3 days. Cell number was counted, and the relative impact of the drug was normalized for siNS or siKLF4 in the absence of the drug. For each graph, nonlinear regression analysis was performed on IC_50_ values of control versus experimental group, and each comparison resulted in statistical significance at *p* < 0.0001. Error bars represent the standard deviation. Experiments were performed three independent times in triplicate

Since KLF4 regulates the expression and downstream activity of EGFR in TNBC cells, we postulated that it may dictate sensitivity to EGFR inhibitors in these cells. Erlotinib is an ATP competitive TKI that binds EGFR and prevents the activation of its downstream signaling cascades including AKT and ERK [44]. To assess its impact on erlotinib sensitivity, KLF4 was overexpressed in MDA-MB-231 cells and its affect on the erlotinib dose-response relationship was assessed by quantifying the number of live cells after 3 days of drug exposure. Dose-response curves were normalized to values obtained with AdKLF4 or AdGFP in the absence of drugs to ensure a specific assessment of efficacy independent of the baseline growth inhibitory effects of AdKLF4. This analysis revealed that enforced expression of KLF4 decreased the IC_50_ for erlotinib from 13.6 to 2.8 μM in MDA-MB-231 cells (Fig. 6a). Similarly, KLF4 overexpression in MDA-MB-468 cells also shifted the dose-response curve to the left, reducing the IC_50_ from 11.4 to 2.6 μM (Fig. 6b). In a complementary experiment, silencing KLF4 expression in MCF10A cells increased the IC_50_ from 1.4 to 11.7 μM (Fig. 6c). Thus, high KLF4 expression sensitizes breast epithelial cells to the growth-suppressive effects of EGFR inhibitors. Moreover, reducing KLF4 expression promotes resistance to such drugs. This is because both KLF4 and erlotinib individually decrease EGFR activity, thus combining the two further sensitizes the cells to erlotinib. These data suggest that KLF4 may play a pivotal role in defining which TNBCs may respond to EGFR inhibitors and future studies should examine the impact of KLF4 on the efficacy of other EGFR inhibitors such as gefitinib or afatinib.

## Discussion

TNBC is associated with worse outcomes compared to other breast cancer subtypes, and EGFR overexpression is common in this disease subtype [45], supporting the potential utility of therapies that target this receptor tyrosine kinase. However, while a small population of recurrent breast cancers do respond to erlotinib, this and other EGFR inhibitors have had low overall success in clinical trials of advanced disease [20–22, 46]. Improving our understanding of drivers that control EGFR expression and response to inhibitors should improve the efficacy of targeting EGFR in TNBC. Here, we report that KLF4 is a negative regulator of metastatic phenotypes (migration, invasion, and proliferation) that functions, in part, by suppressing *EGFR* gene expression. This, in turn, causes a reversal of the same phenotypes that are typically induced by EGFR. Though KLF4 and EGFR are both widely studied in breast cancer [25, 30, 31, 47–49], this is the first report linking their activities in this disease. Our study suggests that KLF4 expression may dictate the efficacy of EGFR inhibitors in TNBC patients. Examining TCGA data did not reveal an association between *KLF4* and *EGFR* mRNAs. However, it is important to note that our studies have found that KLF4 protein regulates the *EGFR* gene. Thus, further analysis using patient data will be necessary to determine whether the relationship between KLF4 protein and *EGFR* mRNA levels are indicative of patient outcomes. Although The Cancer Genome Atlas includes reverse phase protein array (RPPA) data, KLF4 protein is not included within this array, impeding the ability to assess this association. While our studies indicate that KLF4 represses the expression of *EGFR*, Liu and colleagues have recently reported that KLF4 can stimulate *EGFR* gene expression in hepatocellular carcinoma cells [50]. Together, these studies further reinforce the concept that the role of KLF4 is highly context-dependent, acting as an activator or repressor of specific genes depending upon cell lineage.

Previous studies have also shown that other KLF family members can regulate *EGFR*. Like KLF4, KLF5 exhibits a context-dependent role. In primary squamous epithelial cells, KLF5 binds and upregulates *EGFR*, leading to the activation of the MEK/ERK signaling pathway and promoting proliferation [51]. In contrast to KLF4, KLF8 induces invasion, proliferation, and metastasis in breast cancer [52, 53], and in human breast cancers, KLF8 and EGFR are co-elevated [52]. Additionally, KLF8 directly induces *EGFR* gene expression and activates the MEK/ERK pathway in breast cancer to promote invasion and proliferation [53]. To further activate EGFR, KLF8 also represses *miR141*, a microRNA that inhibits translation of EGFR.

Similar to our observations of KLF4 action in TNBC cells, KLF6 is a tumor suppressor that inhibits metastasis and the EGFR/AKT pathway in melanoma and lung adenocarcinoma [54, 55]. In melanoma, KLF6 overexpression reduced the expression of EGFR protein [54]. Furthermore, KLF6 downregulation was correlated with activated EGFR and phosphorylated AKT in patient-derived lung adenocarcinoma samples and decreased KLF6-induced resistance to erlotinib both in vitro and in vivo [55]. In contrast to the role of KLF4 in proliferation and the absence of an impact on apoptosis described herein, KLF6 expression was necessary for erlotinib to induce apoptosis [55]. Notably, this work also revealed a feedback loop wherein EGFR also reduces the expression of KLF6 [55]. Whether EGFR can also control KLF4 expression in TNBC remains to be determined.

We previously identified E-cadherin (CDH1) as a direct transcriptional target of KLF4 [29]. This regulation is required to maintain normal mammary epithelial cell phenotypes, whereas overexpression of KLF4 in MDA-MB-231 cells is sufficient to promote CDH1 expression and reduce invasion and migration. Moreover, the expression of KLF4 was sufficient to inhibit metastatic progression, in vivo [26]. In similar studies, Tiwari et al. identified KLF4 as a central regulator of EMT, with KLF4 transcriptionally activating epithelial genes (including *CDH1* and *END1*) and transcriptionally repressing mesenchymal genes such as *VIM*, *CTNNB1*, and *CDH2* [32]. Accordingly, loss of KLF4 resulted in the induction of EMT programs in breast cancer cells. Thus, these studies in combination with the data presented in this manuscript highlight the crucial function of KLF4 to simultaneously activate and repress gene targets in a coordinated fashion to suppress aggressive breast cancer properties. In addition to EGFR, other promising KLF4 targets were revealed by the RPPA analysis. One of these is neuregulin (*NRG1*), a cell adhesion molecule and a member of the neuregulin family known to regulate the EGFR family [56]. This protein is a ligand of EGFR and promotes proliferation in ovarian and colon cancer models [57, 58]. In breast cancer, EGFR silencing decreased NRG1 expression and invasion, while NRG1 silencing decreased EGFR expression and proliferation [59]. Another study found the NRG1/EGFR interaction to be critical for the promotion of proliferation in breast cancer cells [60]. However, additional studies are necessary to determine if KLF4 also directly regulates *NRG1* expression in breast cancer. The overlapping impact of NRG1 and EGFR on aggressive phenotypes and their feedback regulation of each other suggest that dual inhibition of these proteins may be effective in TNBC. Thus, it may be useful to combine erlotinib with an NRG1 inhibitor. We postulate that the level of synergy that may be observed would be dependent upon the KLF4 expression since EGFR appears to be suppressed by this transcriptional regulator. In addition to NRG1, it is also feasible that additional transcriptional targets of KLF4 contribute to the response to EGFR inhibitors. Hence, the RPPA data presented herein provides a resource for potential mediators that should be further explored.

## Conclusion

In summary, the studies presented herein affirm the ability of KLF4 to suppress pro-metastatic phenotypes in TNBC and uncover a new signaling axis that may explain why EGFR inhibitors have had limited success in treating this disease. Additional studies are required to determine if tumors that do respond to erlotinib are KLF4 positive, as well as to identify other proteins that further predict erlotinib sensitivity in TNBC patients. It is plausible that tumors with high KLF4 expression may be more responsive to anti-EGFR therapies such as erlotinib, making KLF4 a potential biomarker of response

## Supplementary information

**Additional file 1: Figure S1.** KLF4 represses migration, invasion, and cell growth in breast epithelial cells. a REVERT staining of total protein across eight different breast cell lines: MCF10A (10A), MCF7, T47D, SKBR3, SUM159, HCC70, MDA-MB-468 (468), and MDA-MB-231 (231). b REVERT staining of total protein in MCF10A cells as well as MDA-MB-231 and MDA-MB-468 after transduction with AdGFP (G) or AdKLF4 (K). c REVERT staining of total protein in MCF10A cells after transfection with siNS or siKLF4 (siK).

**Additional file 2: Figure S2.** Identification of a KLF4-regulated protein signature reveals EGFR as a downstream target. The top a 63 downregulated proteins and b 14 upregulated proteins after KLF4 overexpression. c Protein-Protein interaction network including proteins with greater than 2-fold expression decrease after AdKLF4 infection and RPPA analysis. Colored nodes indicate query proteins and the first shell of interactors. Teal and purple lines indicate known interactions. Green, red, yellow and blue lines indicate predicted interactions. d REVERT staining of total protein in MDA-MB-231 cells after transduction with AdGFP (AdG) or AdKLF4 (AdK).

**Additional file 3: Figure S3.** KLF4 negatively regulates the EGFR signaling pathway. a REVERT staining of total protein in Fig. 3a. b REVERT staining of total protein in Fig. 3c. c REVERT staining of total protein in Fig. 3e.

**Additional file 4: Figure S4.** Repression of EGFR is an obligatory intermediate step for KLF4 to inhibit aggressive breast cancer phenotypes. a REVERT staining of total protein in Fig. 5a. b REVERT staining of total protein in Fig. 5b.

**Additional file 5: Table S1.** ChIP-PCR primer sequences. Primer sequences targeting six regions within the *EGFR* promoter are listed.

## Data Availability

All data generated or analyzed during this study are included in this published article and its supplementary information files.
