# Supplementary figures and images for: KLF4 defines the efficacy of the epidermal growth factor receptor inhibitor, erlotinib, in triple-negative breast cancer cells by repressing the EGFR gene
Source: Breast Cancer Res. 2020 Jun 18;22:66. doi: 10.1186/s13058-020-01305-7 (PMC7301986; doi:10.1186/s13058-020-01305-7)

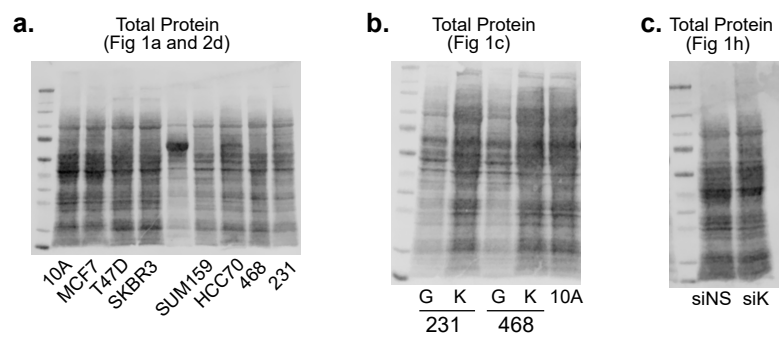

Figure S1

Supplement: Supplementary file 1 — Additional file 1: Figure S1. KLF4 represses migration, invasion, and cell growth in breast epithelial cells. a REVERT staining of total protein across eight different breast cell lines: MCF10A (10A), MCF7, T47D, SKBR3, SUM159, HCC70, MDA-MB-468 (468), and MDA-MB-231 (231). b REVERT staining of total protein in MCF10A cells as well as MDA-MB-231 and MDA-MB-468 after transduction with AdGFP (G) or AdKLF4 (K). c REVERT staining of total protein in MCF10A cells after transfection with siNS or siKLF4 (siK). [file 13058_2020_1305_MOESM1_ESM.pdf]

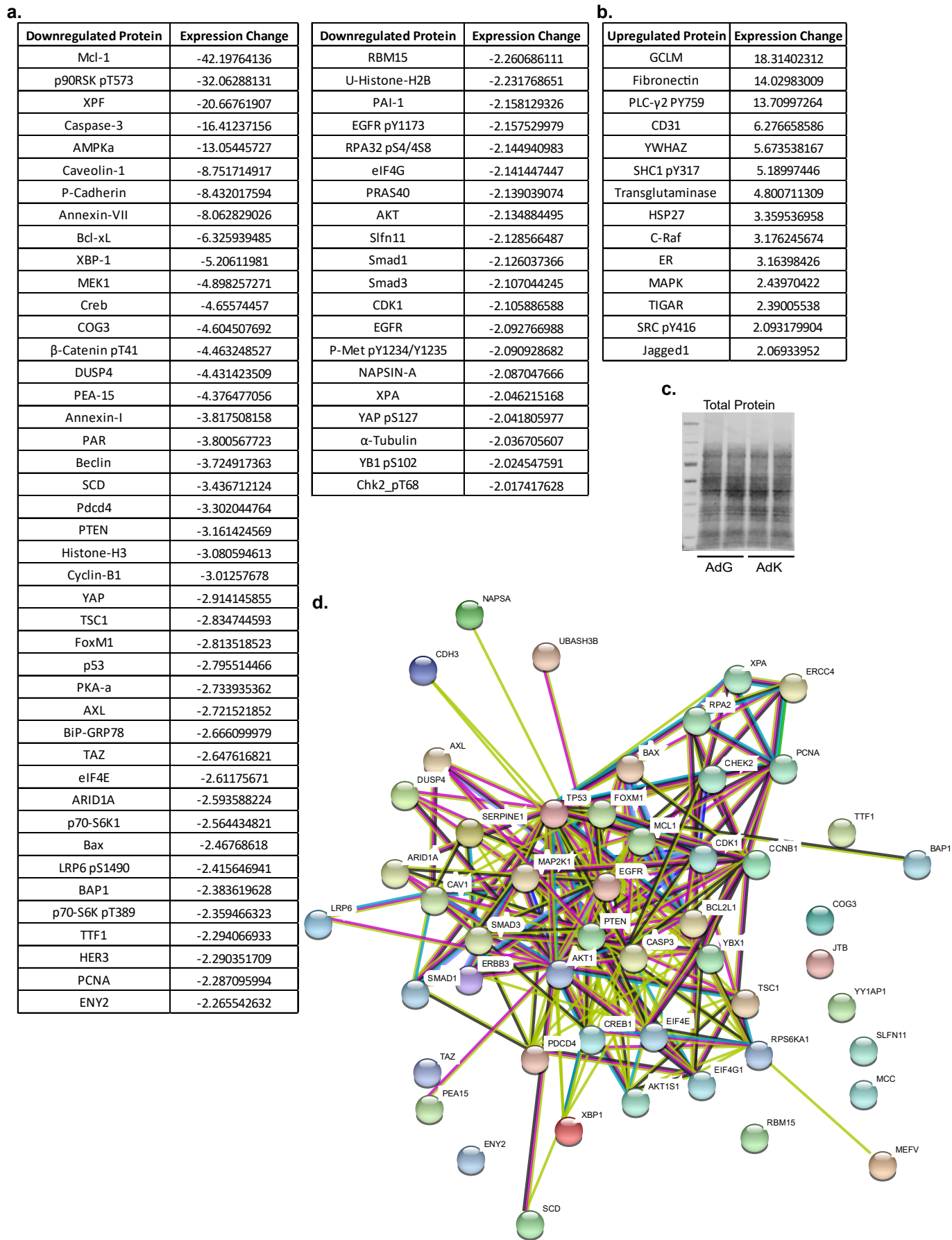

Figure S2

Supplement: Supplementary file 2 — Additional file 2: Figure S2. Identification of a KLF4-regulated protein signature reveals EGFR as a downstream target. The top a 63 downregulated proteins and b 14 upregulated proteins after KLF4 overexpression. c Protein-Protein interaction network including proteins with greater than 2-fold expression decrease after AdKLF4 infection and RPPA analysis. Colored nodes indicate query proteins and the first shell of interactors. Teal and purple lines indicate known interactions. Green, red, yellow and blue lines indicate predicted interactions. d REVERT staining of total protein in MDA-MB-231 cells after transduction with AdGFP (AdG) or AdKLF4 (AdK). [file 13058_2020_1305_MOESM2_ESM.pdf]

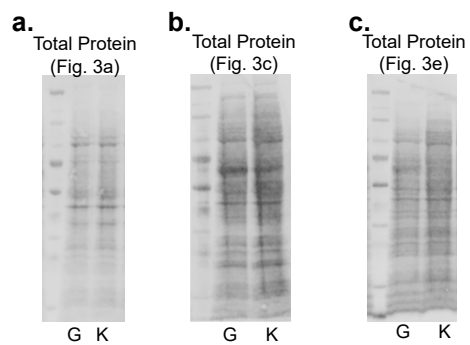

Figure S3

Supplement: Supplementary file 3 — Additional file 3: Figure S3. KLF4 negatively regulates the EGFR signaling pathway. a REVERT staining of total protein in Fig. 3a. b REVERT staining of total protein in Fig. 3c. c REVERT staining of total protein in Fig. 3e. [file 13058_2020_1305_MOESM3_ESM.pdf]

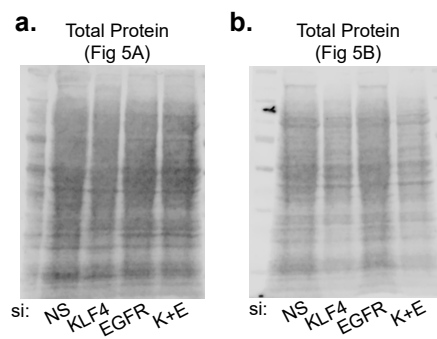

Figure S4

Supplement: Supplementary file 4 — Additional file 4: Figure S4. Repression of EGFR is an obligatory intermediate step for KLF4 to inhibit aggressive breast cancer phenotypes. a REVERT staining of total protein in Fig. 5a. b REVERT staining of total protein in Fig. 5b. [file 13058_2020_1305_MOESM4_ESM.pdf]
