# Supplementary material for: KLF4 defines the efficacy of the epidermal growth factor receptor inhibitor, erlotinib, in triple-negative breast cancer cells by repressing the EGFR gene
Source: Breast Cancer Res. 2020 Jun 18;22:66. doi: 10.1186/s13058-020-01305-7 (PMC7301986; doi:10.1186/s13058-020-01305-7)
Supplement: Supplementary file 5 — Additional file 5: Table S1. ChIP-PCR primer sequences. Primer sequences targeting six regions within the EGFR promoter are listed. [file 13058_2020_1305_MOESM5_ESM.pdf]

| Site | Sequence | Position | Primer Set | Primer Sequence |
|---|---|---|---|---|
| 1 | 5'-ACACACCCAGC-3' | chr7: 55016867 | A/B | F 5’-TGCTTCACACATTGGCTTCA-3’<br>R 5’-TGCTTCACACATTGGCTTCA-3’ |
| 2 | 5'-GACCACCCTCC-3' | chr7: 55016900 | | |
| 3 | 5'-GCACACCCCGA-3' | chr7: 55017840 | C/D | F 5’-GAAGTTCCAGGTTGTGCGG-3’<br>R 5’-CCATCCCCACTGTTCCTTCT-3’ |
| 4 | 5'-ATGCACCCGAC-3' | chr7: 55018092 | | |
| 5 | 5'-CTGCACCCGGA-3' | chr7: 55018363 | E/F | F 5’-TATCCTGGTCACTTCTCCCG-3’<br>R 5’-TGGCTAGTACCTGGAGTGTG-3 |
| 6 | 5'-TGGCACCCCTG-3' | chr7: 55018460 | | |

Table S1
